# Predictors of Acquired Perforating Dermatosis in Uremic Patients on Hemodialysis: A Case-Control Study

**DOI:** 10.1100/2012/158075

**Published:** 2012-12-11

**Authors:** Cheng-Hao Weng, Ching-Chih Hu, Shir-Hwa Ueng, Chun-Chen Yu, Chung-Yee Hui, Ja-Liang Lin, Chih-Wei Yang, Cheng-Chieh Hung, Ching-Wei Hsu, Tzung-Hai Yen

**Affiliations:** ^1^Department of Nephrology, Chang Gung Memorial Hospital, 5 Fu-Shin Street, Kwei-Shan, Taoyuan 333, Taiwan; ^2^College of Medicine, Chang Gung University, Taoyuan 333, Taiwan; ^3^Department of Hepatogastroenterology and Liver Research Unit, Chang Gung Memorial Hospital, Keelung 204, Taiwan; ^4^Department of Pathology, Chang Gung Memorial Hospital, Linkou 333, Taiwan; ^5^Department of Dermatology, Chang Gung Memorial Hospital, Linkou 333, Taiwan

## Abstract

*Objectives*. The purpose of this study was to identify the predictors of acquired perforating dermatosis (APD) in patients on maintenance hemodialysis (HD). *Methods*. A case-control study was performed at our outpatient HD center between January 1, 2000 and March 31, 2011. Patients on HD with APD were matched (1 : 2) for gender and age with controls on HD. Conditional logistic regression was used to identify factors associated with APD. *Results*. A total of 19 patients with APD and 38 age and gender matched patients were enrolled in the study. Univariate logistic regression showed that APD was significantly associated with diabetes mellitus (DM), reduced levels of intact parathyroid hormone (iPTH) and albumin (Alb), reduced dialysis adequacy (*Kt*/*V*) and urea reduction rate (URR), and elevated levels of high-sensitivity C-reactive protein (hsCRP). Multivariate logistic regression identified reduced iPTH (hazard ratio (HR): 0.983; *P* = 0.026) and Alb (HR: 0.099; *P* = 0.047) and elevated hsCRP (HR: 1.210, *P* = 0.024) as risk factors for APD. *Conclusions*. iPTH, hsCRP, and Alb are predictors for APD in HD patients.

## 1. Introduction

Perforating dermatoses are characterized by transepidermal elimination of dermal material (collagen, elastic tissue, or necrotic connective tissue). APD is a major perforating disorder characterized by severely pruritic follicular hyperkeratotic papules, which are sometimes umbilicated, on the hair-bearing limbs of adults [1, 2]. The predictors of APD are diabetes mellitus (DM), chronic kidney disease (CKD), and HD [3]. APD affects about 10% of patients on HD. However, previous studies have not identified potential risk factors for APD in patients on maintenance HD. The purpose of this study was to identify potential risk factors that predict development of APD in patients on maintenance HD.

## 2. Materials and Methods

### 2.1. Subjects

This retrospective observational study complied with the guidelines of the Declaration of Helsinki and was approved by the Medical Ethics Committee of Chang Gung Memorial Hospital, a tertiary referral center located in the northern part of Taiwan. Since this study involved a retrospective review of existing data, approval from the Institutional Review Board was obtained, but without specific informed consent from patients. Furthermore, not only were all individual data securely protected (by delinking identifying information from the main data sets) and made available to investigators only but they were also analyzed anonymously. The Institutional Review Board of Chang Gung Memorial Hospital has specifically waived the need for consent. Finally, all primary data were collected according to procedures outlined in epidemiology guidelines that strengthen the reporting of observational studies.

This study, which was conducted from January 1, 2000 to March 31, 2011 included 19 patients with APD, which was identified from a total of 820 maintenance HD patients in our hospital and diagnosed by dermatologists by skin biopsy, who had been on maintenance HD in our outpatient HD center for more than 3 months. Patients with APD were compared with 38 randomly selected patients from 820 maintenance HD patients in our hospital with gender- and age-matched (1 : 2) controls who did not have APD examined by a dermatologist and had been on maintenance HD for more than 3 months. Demographic and medical data were obtained by chart reviews and from the patient database at our hospital. The values of blood tests were the means of 6 months before the diagnosis of APD was made in cases and at the end of this study in controls. Dialysis adequacy (*Kt*/*V*) was calculated by single pool method. *Kt*/*V* is defined as the dialyzer clearance of urea (*K*, obtained from the manufacturer in mL/min and periodically measured and verified by the dialysis team) multiplied by the duration of the dialysis treatment (*t*, in minutes) divided by the volume of distribution of urea in the body (*V*, in mL), which is approximately equal to the total body water. The *Kt*/*V* is affected by the equilibration of urea from skeletal muscle to plasma water. Urea-rich blood in the venous circulation is not actually measured with the postdialysis BUN sample. Instead, this sample consists of arterial blood flowing into the extracorporeal circuit under conditions in which vascular access recirculation is minimized. The *Kt*/*V* calculated from this sample is called the single pool, nonequilibrated *Kt*/*V*. iPTH was measured by two-site immunometric assay.

### 2.2. Statistical Analysis

Standard descriptive statistics were used to describe the study population. Continuous variables were expressed as mean ± standard deviation and were compared using Student's  *t*-test or Wilcoxon rank-sum test, as appropriate. Dichotomous variables were expressed as numbers (percentage) and compared using *χ* ^2^ test. Conditional logistic regression was used to identify factors associated with APD. Variables yielding *P* value < 0.05 by univariate analysis were entered in a forward multivariate logistic regression analysis. Results of both uni- and multivariate analyses were summarized by hazard ration (HR) and respective 95% confidence interval (CI). Discrimination was evaluated by calculating the area under the receiver operating characteristic curve (AUROC). The cut-off point was calculated by obtaining the best Youden index (sensitivity + specificity-1). Statistical significance was defined as two tailed *P* < 0.05. Analyses were performed with SPSS for Windows Version 12.0 (SPSS, Chicago, IL, USA).

## 3. Ethics

The study protocol was designed in adherence to the Declaration of Helsinki and approved by the Institutional Review Board of our hospital.

## 4. Results

### 4.1. Characteristics of the Study Population

The study population included 19 case patients and 38 control patients. There were no significant differences between the case and control patients with respect to body mass index (BMI), urea reduction ratio (URR), normalized protein catabolic rate (nPCR), time-average urea concentration (TACurea), and levels of hemoglobin (Hb), phosphate (P), potassium (K), total cholesterol (TC), ferritin, and aluminum. There were significant differences in numbers of patients with DM, HD duration, transferrin saturation (TSAT), *Kt*/*V*, and levels of calcium (Ca), iPTH, Alb, triglyceride (TG), and hsCRP (Table 1).

### 4.2. Conditional Logistic Regression Model

Univariate conditional logistic regression showed that APD was significantly associated with DM, reduced iPTH and Alb levels, elevated hsCRP level, and reduced *Kt*/*V* and URR (Table 2). No significant difference in risk was associated with Ca, TG, or TSAT levels. After introducing the patients with DM or not, iPTH, Alb, hsCRP, *Kt*/*V*, and URR values into the forward multivariate conditional logistic regression model, we identified that reduced iPTH (HR: 0.983; *P* = 0.026) and Alb (HR: 0.099; *P* = 0.047) levels and elevated hsCRP levels (HR: 1.210; *P* = 0.024) were risk factors for APD (Table 3).

In patients with DM (*n* = 27), univariate logistic regression showed that reduced iPTH (HR: 0.993; *P* = 0.028) and Alb (HR: 0.209; *P* = 0.033) levels and elevated TG (HR: 1.011; *P* = 0.021) level were associated with APD. Multivariate logistic regression revealed reduced iPTH (HR: 0.987; *P* = 0.022) and Alb (HR: 0.126; *P* = 0.021) levels were risk factors for APD (Table 4).

### 4.3. ROC Curve for iPTH

**Figure 1:**
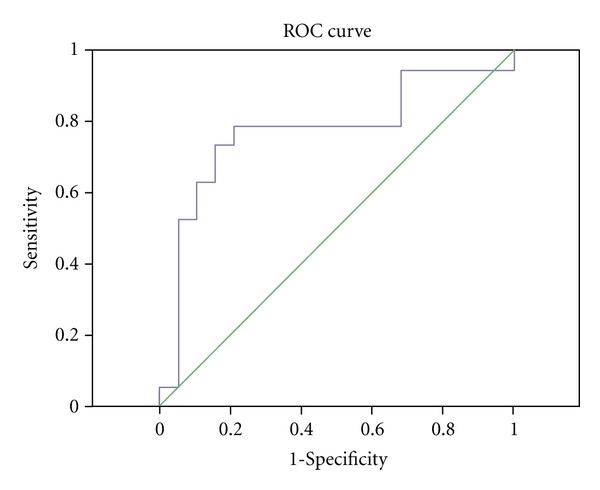
ROC curve for iPTH (AUROC = 0.776 ± 0.067; 95% CI: 0.645–0.906; *P* = 0.001).

It was a novel finding that reduced iPTH level was associated with APD. Computation for the AUROC confirmed the good discriminatory power of the iPTH (AUROC = 0.776 ± 0.067; 95% confidence interval [CI]: 0.645–0.906; *P* = 0.001) (Figure 1). The cut-off point calculated by obtaining the best Youden index was 146.05 pg/mL, with a sensitivity of 73.7% and specificity of 84.2%. An iPTH level lower than 146.05 pg/mL was a good predictor of APD. 

## 5. Discussion

This study is the first to show that iPTH, Alb, and hsCRP levels are associated with APD. In 1986, Andress et al. suggested that iPTH could be a good predictor of osteitis fibrosa in patients undergoing maintenance HD [4]. Analysis of iPTH has become one of the preferred noninvasive tools for assessing renal osteodystrophy (ROD). The National Kidney Foundation Kidney Disease Outcomes Quality Initiative (NKF K/DOQI) Clinical Practice Guidelines for Bone Metabolism and Disease in Chronic Kidney Disease have provided guidance on the use of iPTH to evaluate ROD. A target range of plasma iPTH for stage 5 CKD patients has been suggested to be between 150 and 300 pg/mL [5]. In this study, the mean iPTH level of APD patients was 136.46 ± 33.63 pg/mL. The calculated cut-off value of iPTH to predict APD was found to be 146.05 pg/mL, which was below the physiological level suggested by the NKF K/DOQI guidelines for dialysis patients. In patients on dialysis, the inflection point at which iPTH becomes significantly associated with increased all-cause mortality varied from >400 pg/mL [6] to >600 pg/mL [7]. High iPTH may contribute to the development of left ventricular hypertrophy, hyperlipidemia, insulin resistance, impaired glucose tolerance [8, 9], worsening anemia [10], and immunodeficiency [11]. However, Dukkipati et al. have shown that serum iPTH level below 150 pg/mL was associated with surrogates of malnutrition-inflammation complex in a cohort of 748 maintenance HD patients [12]. Patients with microvasculopathy seem to be highly susceptible to APD [13]. Cheng and colleagues have found that activation of the vascular smooth muscle parathyroid hormone receptor by PTH inhibits Wnt/*β*-catenin signaling, type I collagen protein accumulation, and aortic fibrosis and calcification in diabetic arteriosclerosis [14]. According to this study, an iPTH level below that specified in the NFK K/DOQI guidelines may predispose HD patients to microvasculopathy such as arteriosclerosis, which, in turn, increases the possibility of occurrence of APD.

 DM was a predictor of APD in univariate logistic regression, but not a predictor of APD in multivariate logistic regression. Inaba et al. [15] showed that iPTH secretion may be significantly impaired in HD patients with DM compared with those without DM. Therefore, the effect of DM in predicting APD may be reduced by iPTH in multivariate analysis. But in our study, the predictors of APD in DM patients still included iPTH.

 In our study, hsCRP level was shown to be a predictor of APD. Nagano et al. have recently shown that the tissue level of advanced glycation end products (AGE) is an independent determinant of hsCRP levels in HD patients [16]. Multiple stepwise regression analysis revealed that serum Alb (inversely), tartrate-resistant acid phosphatase 5b (TRAP5b), and skin AGE levels were independent determinants of hsCRP. Further, pulsatility index (PI) was the highest among HD patients with elevated skin AGE and hsCRP levels. The role of AGE in the pathogenesis of APD has recently been noted by Fujimoto et al. [17]. Their results suggest that exposing keratinocytes (KC) to AGE-modified interstitial collagen (types I and III) induces terminal differentiation of KCs via the AGE receptor (CD36), leading to the upward movement of KCs together with glycated collagen. This process is called transepidermal elimination and is a fundamental mechanism in the development of APD. Therefore, hsCRP level is a predictor of APD in HD patients with a positive relationship between hsCRP and peripheral vascular disease [18]. 

 In this study, reduced Alb level was also a predictor of APD. The mean value of Alb level in APD patients was 3.73 ± 0.43 g/dL. There is also good discriminatory power of albumin for APD calculated by AUROC (AUROC = 0.689 ± 0.083; 95% CI: 0.535–0.862; *P* = 0.015). The cut-off point calculated by obtaining the best Youden index was 3.74 g/dL, with a sensitivity of 84.2% and specificity of 58.9%. Previous studied have established that serum Alb < 4 g/dL is inversely and progressively associated with increased risk of death in HD patients [19, 20]. By suppressing synthesis, increasing catabolism and/or vascular permeability to Alb, or a combination of these 2 processes, inflammation is significantly associated with hypoalbuminemia [21]. Reduced iPTH is also associated with malnutrition and inflammation as mentioned previously [12].

A limitation of this study is that the analyses were based on observational data, and, therefore, no causal inference can be made from the study results. Another limitation is the small sample size. Larger multicenter prospective cohort studies are needed.

## 6. Conclusions

This is the first case-control study to identify predictors of APD in HD patients. We have shown that iPTH levels below 150 pg/mL, elevated hsCRP levels, and hypoalbuminemia are predictors for APD in HD patients. Physicians should determine the possible causes of these abnormalities and correct them.

## Figures and Tables

**Table 1:** Patients' main demographic and laboratory data.

	Case	Control	*P* value
Age (year)	52.47 ± 11.99	52.68 ± 12.11	0.95
Male	10 (52.6%)	20 (52.6%)	1.0
DM	13 (68.4%)	14 (36.8%)	0.047
HD duration (year)	5.18 ± 3.75	8.23 ± 7.17	0.04
Hb (g/dL)	11.25 ± 1.31	10.68 ± 0.62	0.085
Hct (%)	34.45 ± 3.91	32.91 ± 2.16	0.123
Ca (mg/dL)	10.13 ± 0.82	9.75 ± 0.51	0.035
P (mg/dL)	5.13 ± 1.49	5.01 ± 0.89	0.702
iPTH (pg/mL)	136.46 ± 33.63	299.4 ± 32.93	0.001
Alb (g/dL)	3.73 ± 0.43	4.03 ± 0.24	0.01
K (mEq/L)	4.69 ± 0.82	4.78 ± 0.53	0.644
TC (mg/dL)	178.86 ± 44.76	176.37 ± 42.94	0.84
TG (mg/dL)	244.17 ± 167.82	136.52 ± 75.88	0.001
AST (U/L)	22.07 ± 12.19	19.31 ± 10.91	0.437
ALT (U/L)	19.41 ± 13.29	15.79 ± 8.14	0.219
Ferritin (ng/mL)	210.59 ± 216.22	279.12 ± 235.89	0.212
TSAT	0.21 ± 0.09	0.27 ± 0.086	0.026
BMI (Kg/m^2^)	22.19 ± 2.27	22.92 ± 4.64	0.664
*Kt*/*V*	1.68 ± 0.28	1.82 ± 0.31	0.026
URR	0.75 ± 0.06	0.78 ± 0.05	0.077
hsCRP (mg/L)	14.72 ± 3.71	3.30 ± 0.53	0.000
nPCR (g/kg/day)	1.23 ± 0.21	2.23 ± 4.03	0.136
TACurea (mg/dL)	43.44 ± 18.37	41.16 ± 7.44	0.740
Al (ug/dL)	1.41 ± 0.79	1.31 ± 0.46	0.695

Abbreviations: nPCR: normalized protein catabolic rate.

TACurea: time-averaged concentration of blood urea nitrogen.

**Table 2:** Univariate analyses of characteristics associated with APD.

	HR	*P* value	95% CI
DM	4.510	0.003	1.651–12.319
iPTH (pg/mL)	0.991	0.000	0.987–0.996
Alb (g/dL)	0.107	0.001	0.030–0.379
hsCRP (mg/L)	1.040	0.002	1.015–1.066
*Kt*/*V*	0.128	0.025	0.021–0.771
URR	0.000	0.019	0.000–0.074

**Table 3:** Multivariate analyses of characteristics associated with APD.

	HR	*P* value	95% CI
iPTH (pg/mL)	0.983	0.026	0.968–0.998
Alb (g/dL)	0.099	0.047	0.01–0.966
hsCRP (mg/L)	1.210	0.024	1.025–1.430

**Table 4:** Multivariate analyses of characteristics associated with APD in DM patients (*n* = 27).

	HR	*P* value	95% CI
iPTH (pg/mL)	0.987	0.022	0.975–0.998
Alb (g/dL)	0.126	0.021	0.022–0.731
